# Supplementary material for: Nascent protein retention at polysomes reduces kinetic barriers to self-assembly
Source: bioRxiv. 2026 May 19:2026.05.17.725791. Preprint. [Version 1] doi: 10.64898/2026.05.17.725791 (PMC13228454; doi:10.64898/2026.05.17.725791)

bioRxiv preprint doi: https://doi.org/10.64898/2026.05.17.725791; this version posted May 19, 2026. The copyright holder for this preprint (which was not certified by peer review) is the author/funder, who has granted bioRxiv a license to display the preprint in perpetuity. It is made available under a CC-BY 4.0 International license.

Supplemental Figure 1: Proteasome inhibition suppresses Aβ42 amyloid nucleation independent of fluorescent tagging, compensatory autophagy, or chaperone upregulation. (A) Representative DAmFRET plot of HEK293T cells expressing mEos-tagged Aβ42 or Aβ40 showing Aβ42 forms a discontinuous DAmFRET profile, characteristic of nucleation-limited phase transitions, including amyloids, while Aβ40 exhibits a single low-AmFRET population. (B) Quantification of amyloid formation via DAmFRET-gating following treatment with diverse classes of proteasome inhibitors. HEK293T cells expressing mEos-Aβ42 were allowed to express the construct for 24 hours prior to a 20-hour treatment with vehicle (Control), 500 nM Bortezomib, 10 μM MG132, 20 μM Lactacystin, or 5 μM Carfilzomib. Broad inhibition of amyloid nucleation across all tested compounds confirms the effect is driven by

bioRxiv preprint doi: https://doi.org/10.64898/2026.05.17.725791; this version posted May 19, 2026. The copyright holder for this preprint (which was not certified by peer review) is the author/funder, who has granted bioRxiv a license to display the preprint in perpetuity. It is made available under a CC-BY 4.0 International license.

proteasome impairment rather than an off-target activity of Bortezomib. (C) SDD-AGE analysis of cell lysates expressing 3xFLAG-tagged Aβ42. Cells were allowed to express the construct for 24 hours and were subsequently treated with vehicle (–) or 10 μM MG132 (+) for 20 hours. Anti-FLAG immunoblotting confirms that proteasome inhibition substantially reduces amyloid formation, demonstrating that the observed suppression is not an artifact of the bulky mEos fluorescent tag. (D) DAmFRET non-linear regression fits representing the fraction of assembled Aβ42 as a function of protein expression (a.u.). Following 24 hours of expression, HEK293T cells were treated for 20 hours with vehicle (Control), 500 nM Bortezomib (BTZ), 100 μM chloroquine (CQ), 0.5 μM wortmannin (WM), or co-treated with BTZ+CQ or BTZ+WM. The inhibition of autophagy by CQ or WM failed to rescue the BTZ-induced suppression of nucleation, indicating that enhanced autophagic clearance is not responsible for the increased kinetic barrier to assembly. (E) DAmFRET non-linear regression fits for HEK293T cells treated with vehicle, 500 nM BTZ, 20 μM of the broad-spectrum HSP70 inhibitor VER-155008, or a combination of BTZ and VER-155008. Cells expressed mEos-Aβ42 with drug treatment upon transfection and were measured after 24 hours. Blocking the primary axis of the heat shock response did not rescue the BTZ-induced nucleation defect, suggesting that an upregulation of chaperones is unlikely to explain the loss of assembly competence. (F) Representative raw fluorescence images (left) and corresponding single-particle tracking trajectory overlays (right) of 50 nm Genetically Encoded Multimeric nanoparticles (GEMs) expressed in HEK293T cells. Cells were treated with either a vehicle control or 500 nM Bortezomib (BTZ) for 20 hours. High-speed time-lapse imaging was performed with a temporal resolution of 10 ms per frame (dt = 10 ms). (G) Quantification of the effective diffusion coefficient (D_{eff}) evaluated at a 100 ms timescale. Data points represent the median D_{eff} across datasets containing multiple cells and thousands of individual GEM trajectories per condition, with error bars denoting the standard error of the mean (SEM). The slight (<10%) reduction in diffusivity (from ~0.448 to ~0.415 μm²/s) upon BTZ treatment indicates that the observed suppression of amyloid nucleation is not driven by a global increase in macromolecular crowding. (H) Immunoprecipitation (IP) validation of mEos-tagged proteins. HEK293T cells were transfected and concurrently treated with vehicle or 500 nM bortezomib (BTZ) for 24

bioRxiv preprint doi: https://doi.org/10.64898/2026.05.17.725791; this version posted May 19, 2026. The copyright holder for this preprint (which was not certified by peer review) is the author/funder, who has granted bioRxiv a license to display the preprint in perpetuity. It is made available under a CC-BY 4.0 International license.

hours. Total cell lysates (Input), IP fractions (Anti-mEos), and depleted lysates (Flow Through) were analyzed by immunoblotting. The arrow denotes the successful enrichment of the ~26 kD target band across conditions. (I) Kyoto Encyclopedia of Genes and Genomes (KEGG) pathway enrichment analysis of significantly downregulated proteins identified via mass spectrometry following BTZ treatment. The lollipop chart displays top-ranked pathways filtered by adjusted *P* value. The x-axis represents fold enrichment (the percentage of list genes in a pathway divided by the corresponding percentage in the background gene set). Node color indicates statistical significance (-log10(FDR), adjusted via the Benjamini-Hochberg method), and node size corresponds to the number of mapped genes overlapping with the target pathway. (J) Pathway interaction network plot illustrating biological overlap between the enriched KEGG pathways. Nodes represent individual pathways, with node sizes scaled to the total number of genes in the respective gene set. Edges connect nodes that share ≥20% of their mapped genes, highlighting highly interconnected biological networks. (K) Hierarchical clustering tree (dendrogram) summarizing the correlation among significantly enriched pathways. Pathways are clustered based on the proportion of shared genes, revealing overarching biological themes systematically suppressed by BTZ treatment. Raw *P* values, calculated using a hypergeometric test, are annotated adjacent to each pathway. All data represent biological triplicates (*n=3*), with error bars indicating mean ± SEM (*$p < 0.05$, $p < 0.01$, ***$p < 0.001$, ****$p < 0.0001$).

Supplementary Figure 2: Translation controls kinetic barriers to self-assembly broadly. (A) Kozak perturbation controls the kinetic and not thermodynamic barrier to formation of protein self-assembly. (Left) Montage of representative DAmFRET plots with spline fit overlays from yeast expressing NLRP3 PYD-mEos showing the Kozak dependence of nucleation and (Right) comparable equilibrium phase transition when seeded ectopically with μNS-NLRP3 PYD. (B) Kozak effect of nucleation is independent of the fluorescent tag. DAmFRET fits showing qualitatively similar effect of Kozak strength while tagged with a smaller fluorogenic tag, frFAST, orthogonal to the mEos-DAmFRET setup. (C) Translation-mediated control of nucleation does not depend on co-translational ribosome quality control signaling. Comparison of fraction of

bioRxiv preprint doi: https://doi.org/10.64898/2026.05.17.725791; this version posted May 19, 2026. The copyright holder for this preprint (which was not certified by peer review) is the author/funder, who has granted bioRxiv a license to display the preprint in perpetuity. It is made available under a CC-BY 4.0 International license.

nucleated polymers in overlapping expression bins show the persistence of both, the Kozak effect as well as that of codon composition of the C-terminal extension in wild-type (WT) and rqc2 (RQC2 deletion strain) (not significant by Two-Way ANOVA).

Supplemental Figure 3: Computational analysis of translation efficiency of IDRs in the human proteome shows co-translation interactions potentially controls IDR behavior. (A) Mean predicted TE via RiboNN showing a characteristic negative dependence on protein length. (B) Comparison of first (Bottom 25%, attractive) and last (Top 25%, repulsive) quartiles by Epsilon score (net self-attractiveness) showing elevated TE for the former, explained by attractive IDRs being present in shorter proteins. (C-E) Data represents the exact content of main Figure 5 A-C, but for the alternate computed self-interaction score (Epsilon2).

bioRxiv preprint doi: https://doi.org/10.64898/2026.05.17.725791; this version posted May 19, 2026. The copyright holder for this preprint (which was not certified by peer review) is the author/funder, who has granted bioRxiv a license to display the preprint in perpetuity. It is made available under a CC-BY 4.0 International license.

Figure S1

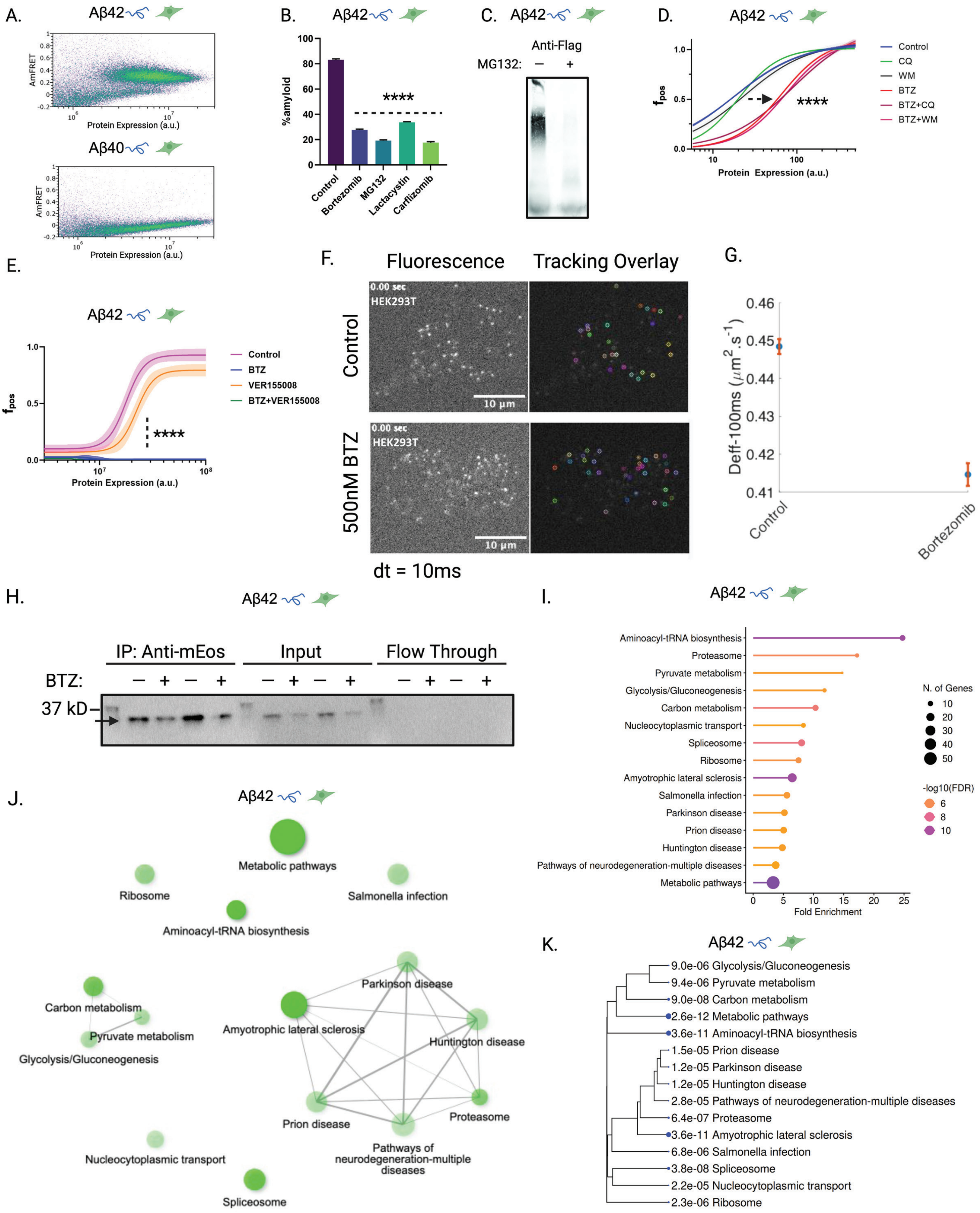

bioRxiv preprint doi: https://doi.org/10.64898/2026.05.17.725791; this version posted May 19, 2026. The copyright holder for this preprint (which was not certified by peer review) is the author/funder, who has granted bioRxiv a license to display the preprint in perpetuity. It is made available under a CC-BY 4.0 International license.

Figure S2

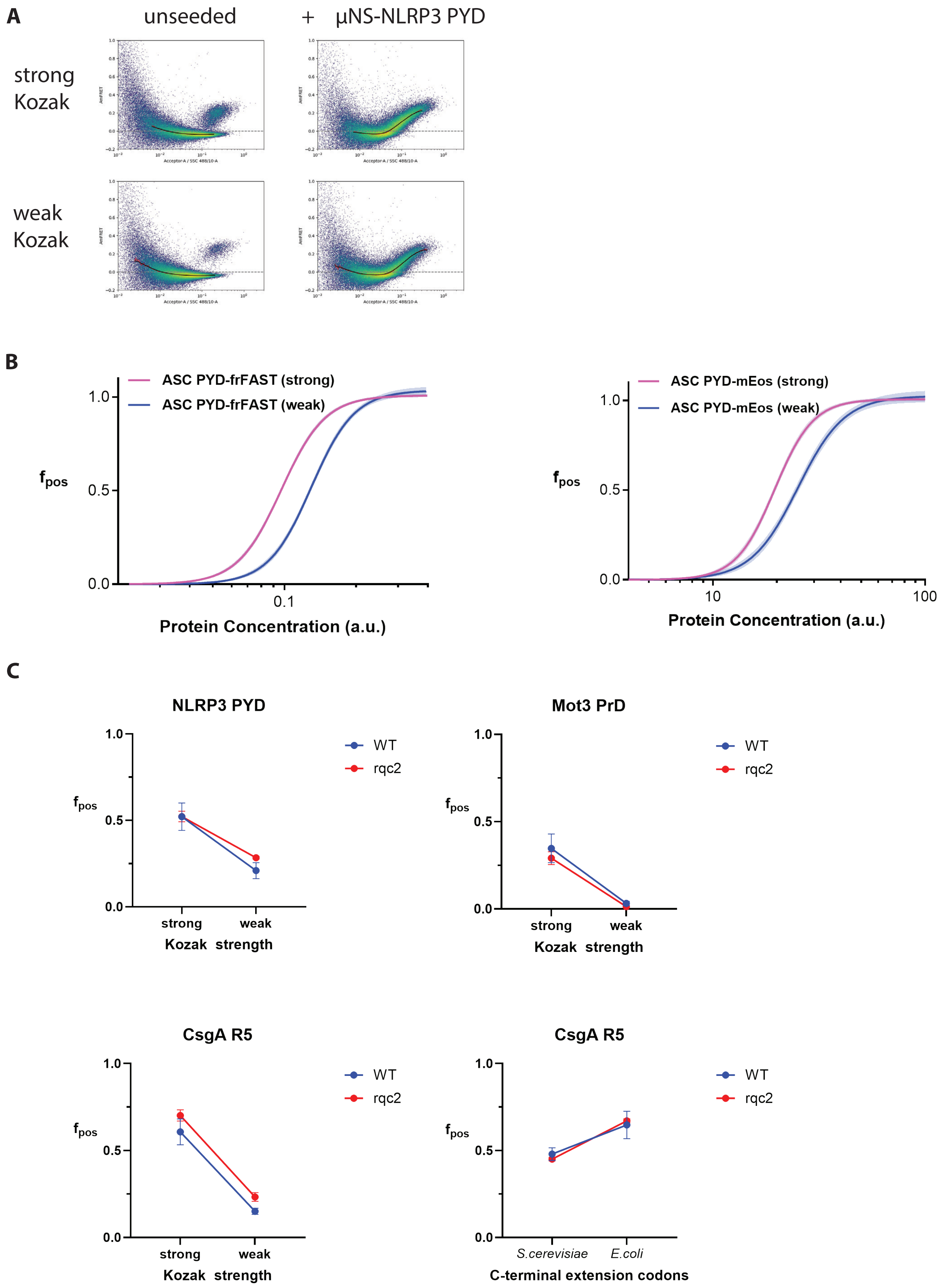

bioRxiv preprint doi: https://doi.org/10.64898/2026.05.17.725791; this version posted May 19, 2026. The copyright holder for this preprint (which was not certified by peer review) is the author/funder, who has granted bioRxiv a license to display the preprint in perpetuity. It is made available under a CC-BY 4.0 International license.

Figure S3

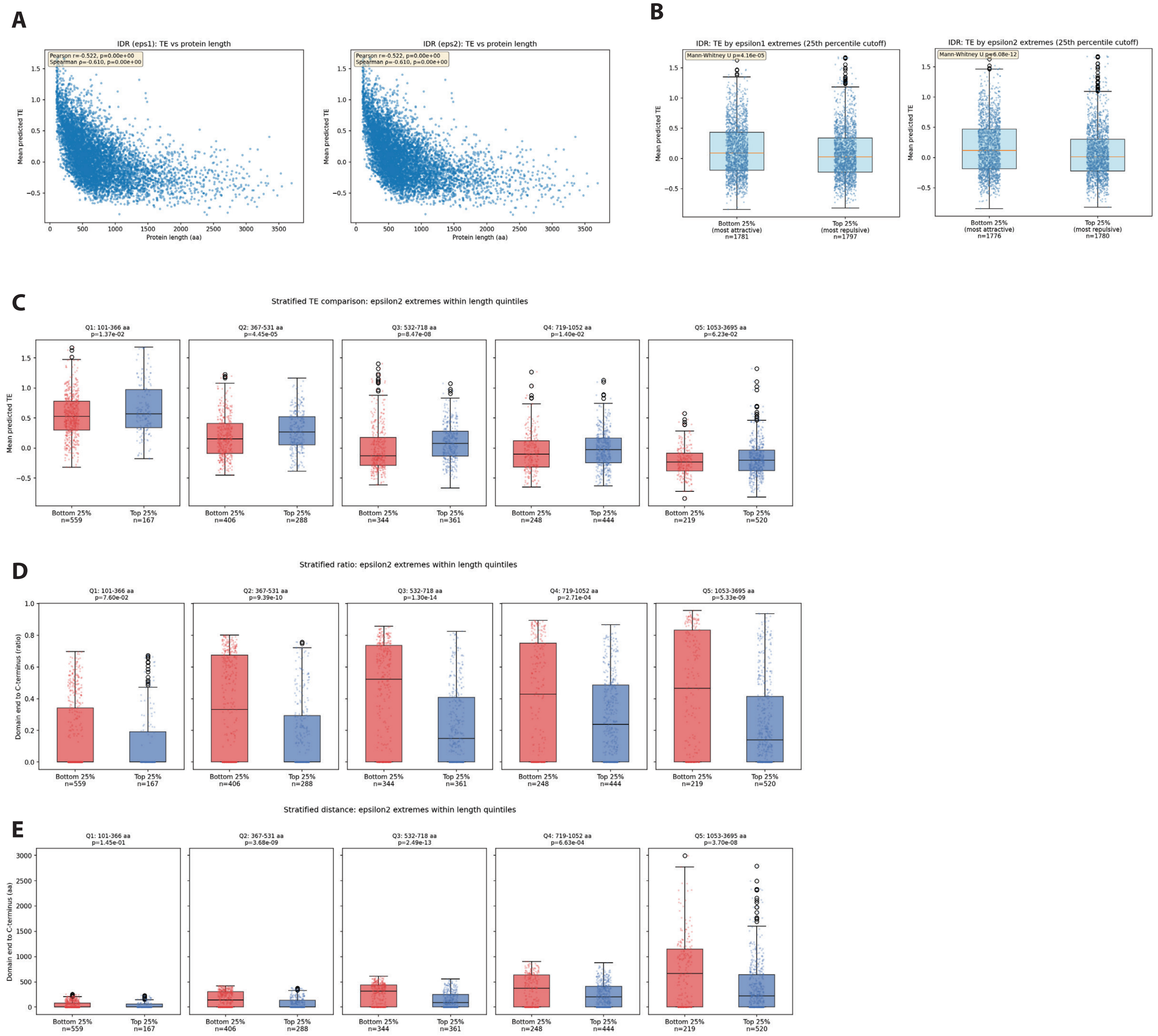

Supplement: Supplement 3 [file NIHPP2026.05.17.725791v1-supplement-3.pdf]
